# Clinical assessment of suicide risk and suicide attempters' self-reported suicide intent: A cross sectional study

**DOI:** 10.1371/journal.pone.0217613

**Published:** 2019-07-05

**Authors:** Carol C. Choo, Keith M. Harris, Peter K. H. Chew, Roger C. Ho

**Affiliations:** 1 College of Healthcare Sciences, James Cook University, Singapore, Singapore; 2 School of Psychology, Charles Sturt University, Port Macquarie, NSW, Australia; 3 School of Psychology, University of Queensland, St Lucia, Queensland, Australia; 4 Yong Loo Lin School of Medicine, National University of Singapore, Singapore, Singapore; 5 Institute for Health Innovation and Technology (iHealthtech), National University of Singapore, Singapore, Singapore; 6 Centre of Excellence in Behavioral Medicine, Nguyen Tat Thanh University (NTTU), Ho Chi Minh City, Vietnam; University of Toronto, CANADA

## Abstract

This study explored medical doctors’ clinical assessment of suicide risk and suicide attempters’ self-reported suicide intent. Three years of archival assessment records related to suicide attempters who were admitted to the emergency department of a large teaching hospital in Singapore were subjected to analysis. Records related to 460 suicide attempters (70.4% females; 28.6% males) were analysed using logistic regressions. Their ages ranged from 12 to 85 (*M* = 29.08, *SD* = 12.86). The strongest predictor of suicide intent was habitual poor coping, followed by serious financial problems, and expressed regret. The strongest predictor of suicide risk was hiding the attempt followed by prior planning. The findings were discussed in regards to implications in clinical assessments and suicide prevention efforts.

## Introduction

Suicide is a serious problem worldwide, with an annual global age-standardized suicide rate of 11.4 per 100 000 population [1]. Suicide attempts are also a serious public health problem, with significant tolls on psychiatric and other healthcare services. Hospitalizations for attempted suicides occur at a rate of six to seven times that of completed suicides [2], and are an important predictor of eventual suicide [3].

A review of articles examining suicidal behaviour highlights the importance of definitional clarity [4]. Suicide attempt is defined as a self-inflicted, potentially injurious behaviour with a non-fatal outcome for which there is evidence of intent to die, and it is differentiated from self-harm, where it is evident that there is no intent to die [5], and highlights the importance of suicide intent in differentiating both behaviours [4,5]. A high degree of suicide intent is associated with hopelessness, pessimism [6], a sense of isolation, older age, a history of suicide attempts, and a higher risk for completed suicide [7].

Suicide attempts have been rated in terms of the medical consequences or medical lethality and seriousness of the intent of the individual to die [8]. The Beck Medical Lethality Scale [9] addresses the medical lethality of the suicide attempt but gathers very little other information. The Suicidal Behaviour Questionnaire [10] asks for a count of previous self-injuries and suicide attempts together with information about the medical treatment for each, but does not include other aspects of the attempt. Other lethality scales which have been developed more recently are more comprehensive, as they include other dimensions in addition to medical severity, and empirical support has been cited [4,11].

Subjective intent to die has been demonstrated to be of high importance, through empirical studies of multiple samples and advanced statistical analyses [12]. Suicide intent could be measured by the Suicide Intent Scale SIS [13], which includes items on the preparation and manner of execution of the attempt, the setting, prior clues given that could facilitate or hamper intervention or discovery, the attempter’s perception of the lethality of the method, the extent of premeditation, purpose, and expectation of the possibility for rescue. Factor analysis of the SIS revealed four factors: attitude towards the attempt, planning, precautions against intervention, and communication with others. Subsequent research [14] found that the precautions dimension differentiated between attempters who did and did not ultimately die by suicide, implying that attempters who took precautions against being discovered, such as those who found isolated places and scheduled their attempts at times when discovery was less likely, would be particularly at risk of eventual suicide. An important component of the nature of suicide is the typical pattern of the index attempt. Those who had taken precautions against discovery at the time of the index attempt might have been more likely to use similar methods at the ultimate attempt. Since precautions would militate against discovery and intervention, this pattern, if repeated, would seem likely to foster a successful suicide.

Suicide intent and lethality may have overlapping features, and most clinicians will assume that high medical lethality suggests high suicide intent. However, lethality may not be a true reflection of intent [15]. Greater suicide intent increases suicide risk [16], but studies did not consistently show an association between suicide intent and lethality of the suicide attempt.

Some researchers found minimal association between the degree of suicide intent and the extent of medical lethality in suicide attempts [9,17,18]. However, for attempters with accurate expectations about the likelihood of dying from the attempts, medical lethality was proportional to the degree of suicide intent. Attempters were more likely to make medically severe attempts when they had accurate expectations of the lethality of the method used, and higher suicide intent [17]. A study done in China found a positive correlation between suicide intent and lethality. Zhang and Xu [19] postulated that the high fatality rate for Chinese females who swallowed poisonous pesticides was a function of strong suicide intent and the well-known lethality of pesticides. In contrast, some studies found that the medical lethality of the chosen method did not match the adolescent attempter’s intent to die, as the adolescent had limited knowledge of the toxicity [20]. It has been suggested that both suicide intent and medical lethality should be assessed when ascertaining the seriousness of the attempt.

Neimeyer and Pfeiffer [21] cited the inadequate assessment of suicide intent as one of the common errors of suicide interventionists. It was suggested that effective assessments should include assessment of suicide intent, lethality of the suicide plan (e.g., by enquiring about precautions against discovery and rescue), and perturbation associated with the suicide plan [7,21].

Issues that have been identified in suicide risk assessment include membership of a high risk group, the acuteness of risk, the need for risk indicators to be clinically relevant, and the recognition that the risk factors are multi-dimensional, intersecting and interacting [22]. Background risk factors include socio-demographic and related indices which are correlated with increased risk for suicide. They can assist the clinician in overall formulation of suicide risk.

Beautrais [23] proposed that in the accumulative risk model, the risk of serious suicide attempts rose dramatically with the risk factor burden to which an individual was exposed. Four or more risk factors were found to elevate the odds of serious suicide attempts over 120 times more than those with fewer than three risk factors. Risk increases as the risk factors accumulate for a suicidal individual [7]. Suicide risk is also increased with certain combinations of risk factors, for example, patients with bipolar disorder and comorbid alcohol use disorder had twice the suicide risk of those with bipolar disorder but without alcohol use disorder [24]. Substantially higher risks occur among those with history of suicide attempts and psychiatric diagnosis, such as mood disorder and schizophrenia [23, 25–27].

When researchers tried to use the commonly recognized suicide risk factors to predict suicide, predictive power was found to be poor with low clinical utility. Pokorny [28] used the 20 best predictors of suicide to identify the 67 subjects who died by suicide in a sample of 4,800 American veterans. Statistical analysis yielded 1,206 false-positive identifications, and had limited usage in a clinical capacity. Goldney and Spence [29] also found that the predictive ability of six clinical features of suicide was poor. Even in high risk patients with affective disorders, prediction of suicide using the suicide risk factors was poor [30]. Furst and Huffine [31] found that when subjects were asked to predict suicide, the potential for suicidal behaviour was under-estimated. Factors associated with accurate prediction were female gender and presence of a family member who died by suicide.

In summary, there are limitations in the usage of checklists on recognized risk factors, and standardized assessments in assessment of suicide risk and intent. There is insufficient evidence to support a model for accurate prediction of suicide risk. An investigation of the current practice in clinical assessment of suicide risk, and factors contributing to suicide intent for suicide attempts in the local context would inform efforts in defining best practice in suicide assessment.

A review of relevant literature shows that many risk and protective factors were related to suicide deaths and suicide attempts in both Western and Asian studies, listed in the next two paragraphs. However, there is a lack of large scale research examining prediction of suicide intent and risk using recognized suicide risk and protective factors, and circumstances surrounding the attempt.

This current study aims to explore prediction of medical doctors’ clinical assessment of suicide risk and suicide attempters’ self-report of suicide intent. Based on past evidence in both Western and Asian studies, analysis will be conducted on the following available variables. These variables were collected as part of standard clinical assessment. The risk factors include: living alone [32], unemployment [32–35], financial problem [36], physical illness [36], mental illness [34,35,37], alcohol/ drug use [38,39] interpersonal conflict [34,35,40] protective factors include: presence of dependents [41], emotional support [42], willingness to seek help [43,44], resolution of precipitants [45], religion [46], regret of the attempt [47], and positive future planning [48]. It is hypothesized that their suicide risk and suicide intent will be predicted by the above-mentioned risk and protective factors as well as features of the attempt, e.g., planning, and precautions taken to hide the attempt [8,14].

## Method

### Procedure

Ethics approval was obtained from the Domains-Specific Review Board of a large teaching hospital in Singapore and the Human Research Ethics Committee at James Cook University. This study is based on an archival retrospective review of de-identified hospital records of patients who were admitted for a suicide attempt from January 2004 to December 2006. Data were collected from the hospital database related to the suicide attempters who were admitted over the three year period and this data set is the most comprehensive data set available from the hospital, as such assessment data were not collected prior to and following the stipulated period. Archival data was extracted from the Patient Psychiatric Assessment Form (PPAF). The PPAF includes the Suicide Risk Assessment Form.

All cases of attempted suicide were assessed by medical doctors in the emergency department under the supervision of a consultant psychiatrist, and the interview took approximately 20 minutes. This assessment was part of the protocol standard operating procedure for patients admitted following a medically treated suicide attempt. At the time of the evaluation, the medical doctor made a formal psychiatric and/ or medical diagnosis. After the assessment, a management plan was recommended.

The inclusion criterion for the current study were suicide attempt cases admitted to the emergency department from January 2004 to December 2006 and were assessed by medical doctors using the PPAF. Data were extracted from multiple hospital databases in relation to the suicide attempt. The majority of them (78.5%) overdosed in the suicide attempt.

### Measures

#### Suicide risk assessment form

The Suicide Risk Assessment Form is a 2-page questionnaire designed to be conducted as a semi-structured interview by medical doctors. The questions used in the semi-structured interview were developed based on consensus from consultant psychiatrists at the hospital who were experienced in suicide risk assessment. This semi-structured interview was devised for the collation of information deemed important for clinical usage in suicide risk assessment and recommendation of management plan, and psychometric properties were not available. The content of the assessment form includes demographic information such as gender, age, and ethnicity. It documents presence of prior planning, efforts to hide the suicide attempt, and suicide attempters’ report of suicide intent, on dichotomous scales (yes and no). It records the presence of risk and protective factors, as well as recommended management plan. The risk factors are recorded on discrete dichotomous scales (yes and no) and include: lack of confidantes, living alone, unemployment, financial problem, mental illness or suicide in the family, alcohol or drug abuse, history of mental illness, interpersonal conflict, and poor coping; the protective factors are recorded on discrete dichotomous scales (yes and no) and include: presence of dependents, emotional support, willingness to seek help, resolution of precipitant, religion, regret, and positive future planning. It records the medical doctor’s clinical assessment of current suicide risk on a 4-point scale (low, low to moderate, moderate to high, high). Suicide risk was recoded, low and low to moderate were recoded into low and moderate and moderate to high were recoded into high.

### Data analysis

The data were analyzed using SPSS version 23 with the alpha level set at .05. Two binary logistic regressions were conducted with suicide intent (Yes versus No) and suicide risk (High versus Low) as the criterion variables, respectively. A total of 20 independent variables were included in each regression: 10 risk factors (e.g., lack of confidantes), 7 protective factors (e.g., has dependants), and 3 features of the suicide attempt (i.e., prior planning, attempt to hide, and place of suicide attempt). The risk and protective factors are listed in Table 1.

**Table 1:** Percentage of patients assessed with high or low suicide risk, risk and protective factors, and suicide intent (*n* = 460).

Predictors and Criteria	Percentage (%)	
	Yes	No
**Risk Factors** 1) Lack of confidantes 2) Living alone 3) Unemployment 4) Serious financial problems 5) Serious physical illness 6) Mental illness/Suicide in family 7) Alcohol/Drug abuse 8) History of Mental illness 9) Ongoing interpersonal conflict 10) Habitual poor coping **Protective Factors** 1) Has dependants 2) Emotional support 3) Willing to seek help 4) Resolution of precipitant 5) Religion 6) Expressed regret 7) Positive future planning **Criteria** 1) Suicide Intent 2) Suicide Risk	35.9 11.1 16.5 14.8 5.0 10.9 17.4 24.8 48.5 36.1 59.3 76.1 70.7 49.1 35.0 81.7 75.2 4.3 4.3 (High)	64.1 88.9 83.5 85.2 95 89.1 82.6 75.2 51.5 63.9 40.7 23.9 29.3 50.9 65.0 18.3 24.8 95.7 95.7 (Low)

## Results

A total of 671 suicide attempt cases were analyzed. Cases with missing data on at least one of the key variables were removed from the data set (*n* = 211), resulting in a sample of 460 cases (70.4% females; 61.7% Chinese, 15.0% Malays, 16.1% Indians, 6.3% Others, and .9% unknown). Their ages ranged from 12 to 85 (*M* = 29.08, *SD* = 12.86).

Medical doctors’ clinical assessment of suicide risk was recoded: ‘low’ and ‘low to moderate’ were recoded into ‘low risk’ whereas ‘moderate to high’ and ‘high’ were recoded into ‘high risk’. This procedure was done because there were insufficient frequencies in the ‘moderate to high’ and ‘high’ categories. The percentages of suicide attempters assessed with suicide risk (High, Low), as well as risk and protective factors, and self-reported suicide intent are presented in Table 1. In addition to risk and protective factors, features of the attempt, such as prior planning (11.3% Yes, 88.7% No), attempt to hide (30.9% Yes, 69.1% No), and place of suicide attempt (80% Home, 2.2% Workplace, 9.3% Public place, 2.8% Friend’s house, .7% Public housing, and 5% Others) were also included in the analysis.

Logistic regression was performed to examine the prediction of self-reported suicide intent. The model contained 20 independent variables (see Table 1). The full model containing all predictors was statistically significant, *χ*2 (24, *N* = 460) = 79.99, *p* < .001, indicating that the model was able to distinguish between suicide attempters with and without suicide intent. The model as a whole explained between 15.9% (Cox and Snell *R*^2^) and 52.9% (Nagelkerke *R*^2^) of the variance in suicide intent, and correctly classified 96.1% of the suicide attempters with suicide intent. As shown in Table 2, only three of the independent variables made a unique statistically significant contribution to the model (serious financial problems, habitual poor coping, and expressed regret). The strongest predictor of suicide intent was habitual poor coping, with an odds ratio of 8.11. This indicated that patients who had habitual poor coping were 8.11 times more likely to be assessed with suicide intent than those who did not have habitual poor coping, controlling for all other predictors in the model. The second strongest predictor was serious financial problems with an odds ratio of 4.39. This indicated that patients who had serious financial problems were 4.39 times more likely to be assessed with suicide intent than those who did not have serious financial problems, controlling for all other predictors in the model. The last predictor was expressed regret with an odds ratio of .09. This indicated that patients who expressed regret were .09 times less likely to be assessed with suicide intent than those who did not express regret, controlling for all other predictors in the model.

**Table 2:** Logistic regression predicting suicide intent (*n* = 460).

Predictors	B	S.E.	Wald	*df*	*p*	OR	95.0% CI for OR	
							Lower	Upper
**Risk Factors** 1) Lack of confidantes 2) Living alone 3) Unemployment 4) Serious financial problems 5) Serious physical illness 6) Mental illness/Suicide in family 7) Alcohol/Drug abuse 8) Mental illness 9) Ongoing interpersonal conflict 10) Habitual poor coping **Protective Factors** 1) Has dependants 2) Emotional support 3) Willing to seek help 4) Resolution of precipitant 5) Religion 6) Expressed regret 7) Positive future planning **Other Variables** 1) Prior planning 2) Attempt to hide 3) Place of act Home vs. Workplace Home vs. Public place Home vs. Friend’s house Home vs. HDB building Home vs. Others Constant	-.33 -.05 -1.34 1.48 1.95 -18.25 1.05 .45 -.54 2.09 -.77 .01 -.63 -1.34 -.24 -2.40 .04 1.19 .22 -18.07 .17 -16.74 -16.62 -.79 -2.66	.74 .92 .88 .72 1.06 4583.89 .71 .69 .68 .90 .68 .78 .67 .96 .70 .79 .72 .79 .70 10702.83 .90 9286.06 21837.76 1.33 1.09	.10 .00 2.33 4.20 3.39 .00 2.18 .44 .64 5.37 1.27 .00 .91 1.96 .12 9.27 .00 2.26 .10 .41 .00 .03 .00 .00 .36 5.96	1 1 1 1 1 1 1 1 1 1 1 1 1 1 1 1 1 1 1 5 1 1 1 1 1 1	.659 .954 .127 .040 .066 .997 .139 .510 .425 .020 .260 .994 .341 .162 .731 .002 .958 .132 .754 .995 .999 .854 .999 .999 .551 .015	.72 .95 .26 4.39 7.04 .00 2.87 1.57 .58 8.11 .47 1.01 .53 .26 .79 .09 1.04 3.30 1.24 .00 1.18 .00 .00 .45 .07	.17 .16 .05 1.07 .88 .00 .71 .41 .15 1.38 .12 .22 .14 .04 .20 .02 .26 .70 .32 .00 .20 .00 .00 .03	3.07 5.79 1.46 18.07 56.29 - 11.59 6.05 2.21 47.58 1.76 4.60 1.96 1.71 3.11 .43 4.22 15.59 4.89 - 6.87 - - 6.11

Logistic regression was performed to examine the prediction of suicide risk. The model contained 20 independent variables (see Table 1). The full model containing all predictors was statistically significant, *χ*2 (24, *N* = 460) = 75.23, *p* < .001, indicating that the model was able to distinguish between patients assessed with high and low suicide risk. The model as a whole explained between 15.1% (Cox and Snell *R*^2^) and 50.1% (Nagelkerke *R*^2^) of the variance in suicide risk, and correctly classified 96.1% of the cases. As shown in Table 3, only two of the independent variables made a unique statistically significant contribution to the model (prior planning and attempt to hide). The strongest predictor of suicide risk was attempt to hide, with an odds ratio of 13.13. This indicated that suicide attempters who tried to hide the attempt were 13.13 times more likely to be assessed as high suicide risk than those who did not attempt to hide, controlling for all other predictors in the model. The second strongest predictor was prior planning with an odds ratio of 8.32. This indicated that suicide attempters who had prior planning were 8.32 times more likely be assessed as high suicide risk than those who did not have prior planning, controlling for all other predictors in the model.

**Table 3:** Logistic Regression Predicting Suicide Risk (*n* = 460).

Predictors	B	S.E.	Wald	*df*	*p*	Odds Ratio	95.0% CI for Odds Ratio	
							Lower	Upper
**Risk Factors** 1) Lack of confidantes 2) Living alone 3) Unemployment 4) Serious financial problems 5) Serious physical illness 6) Mental illness/Suicide in family 7) Alcohol/Drug abuse 8) Mental illness 9) Ongoing interpersonal conflict 10) Habitual poor coping **Protective Factors** 1) Has dependants 2) Emotional support 3) Willing to seek help 4) Resolution of precipitant 5) Religion 6) Expressed regret 7) Positive future planning **Other Variables** 1) Prior planning 2) Attempt to hide 3) Place of act Home vs. Workplace Home vs. Public place Home vs. Friend’s house Home vs. HDB building Home vs. Others Constant	.09 1.38 -.10 .87 -17.83 1.39 1.30 .38 .25 .03 .23 .08 -.54 -.45 -.50 -.99 -.73 2.12 2.58 -18.78 -2.29 -16.47 -14.56 -.70 -4.71	.68 .85 .91 .72 7419.68 .73 .73 .66 .65 .65 .70 .75 .63 .74 .68 .72 .71 .64 .71 9806.64 2.18 10248.96 22962.36 1.23 1.40	.02 2.65 .01 1.48 .00 3.65 3.13 .33 .14 .00 .11 .01 .74 .38 .53 1.90 1.07 11.03 13.25 1.36 .00 1.11 .00 .00 .32 11.32	1 1 1 1 1 1 1 1 1 1 1 1 1 1 1 1 1 1 1 5 1 1 1 1 1 1	.901 .103 .911 .224 .998 .056 .077 .568 .708 .967 .736 .915 .389 .540 .465 .169 .300 .001 < .000 .929 .998 .293 .999 .999 .571 .001	1.09 3.98 .90 2.39 .00 4.00 3.66 1.46 1.28 1.03 1.26 1.08 .58 .64 .61 .37 .48 8.32 13.13 .00 .10 .00 .00 .50 .01	.29 .76 .15 .59 .00 .96 .87 .40 .36 .29 .32 .25 .17 .15 .16 .09 .12 2.38 3.28 .00 .00 .00 .00 .05	4.13 20.98 5.35 9.73 - 16.58 15.38 5.30 4.59 3.67 4.94 4.71 2.00 2.71 2.32 1.52 1.92 29.06 52.5 - 7.21 - - 5.53

*Note*. Suicide Risk was recoded from a 4-point Likert Scale (0 = Low, 1 = Low to Moderate, 2 = Moderate to High, and 3 = High) to a dichotomous variable (0 and 1 = Low, and 2 and 3 = High).

## Discussion

This study aimed to explore the prediction of medical doctors’ clinical assessment of suicide risk and suicide attempters’ self-reported suicide intent. Three years of medical records of 460 suicide attempters were analyzed. It was hypothesized that suicide risk and suicide intent would be predicted by recognised risk and protective factors, as well as features of the attempt.

As hypothesized, the full model containing all the recognized risk and protective factors and features of the attempt was significant in distinguishing suicide attempters with and without suicide intent, correctly classifying 96% of the cases. The strongest predictor of suicide intent was habitual poor coping, followed by serious financial problems, and expressed regret. The association between coping, financial problems and suicide intent may explain the well established relationships between poor coping [49] and financial problems [50,51] with suicides. The concept of regret was a variable collected in the interview, but it is not well established in previous literature. The finding that those who expressed regret were less likely to report suicide intent, might imply that the suicide attempt could be a ‘cry for help’ but not reflective of a desire to die, suggesting that likely interventions include those that promote better decision making and regret regulation [52].

As hypothesized, the full model containing all the recognized risk and protective factors and features of the attempt was significant in distinguishing suicide attempters assessed with high and low suicide risk, correctly classifying 96% of the cases. The strongest predictor for suicide risk was hiding the attempt followed by prior planning. This is consistent with previous literature which reported that attempters who took precautions against being discovered, such as those who found isolated places and scheduled their attempts at times when discovery was less likely, would be particularly at risk of eventual suicide [14].

The results of this study provided evidence to support best practice for suicide assessment and management for medical doctors in the Emergency Department of the acute hospital. The results are consistent with qualitative informal feedback from the hospital’s clinical staff that the semi-structured interview has worked well in that it is brief, and it allows suicide risk assessment to be done quickly without missing important items on suicide risk assessment. When a brief and accurate suicide risk assessment and effective suicide management is needed in a busy clinical environment following a suicide attempt, evidence from this study supports the assessment of prior suicide planning and attempt to hide to be incorporated into the risk assessment protocol. Information about the place of the index attempt could be collected, which might be corroborating evidence for whether precautions might have been taken to reduce the possibility of being discovered and rescued, e.g., attempting suicide alone in one’s home would lessen the probability for discovery by potential persons who could intervene and rescue, as compared to the workplace, friend’s house, public place or public housing. This could be in addition to the assessment of recognised risk and protective factors. Such information could be integrated to inform risk assessment, and to formulate a suicide management plan, together with incorporation of contribution of habitual poor coping, serious financial problems, and expressed regret into suicide management. Previous research had found that brief interventions [53], including safety planning [54] reduced suicide risk post-discharge from the Emergency Department. Results from the current study suggest brief interventions could also include practical strategies to enhance coping. The results also suggest that brief interventions with the suicide attempter could include clarification if there was expressed regret of the attempt, if the attempt was a cry for help and not intent to die, and reasons for living could be elicited.

Limitations of the study include a cross-sectional design, a longitudinal design could enhance the interpretability of the results, as well as including outcomes such as multiple hospital admissions of repeated suicide attempts or eventual suicide death. The criterion variables, including suicide intent were not measured using validated instruments. Future research could employ valid continuous measures of suicidal symptoms. The reliance on self-report and the usage of single dichotomous items also constrains the depth of information obtained. It might be possible for attempters to mask their suicide intent in their self-reports if they feared negative consequences of reporting high-risk behaviours. Future research could employ qualitative interviews to reveal the interplay of relationships and processes impacting on suicide risk, and suicide intent that might be relevant for understanding suicide attempts. In this study, suicide intent was recorded dichotomously and medical doctor’s clinical assessment of current suicide risk was recorded on a 4-point response set, and then recoded into dichotomous variables. Classifying suicide risk and intent as discrete or dichotomous outcomes is often necessary in a busy emergency department to inform clinical decision making and treatment planning. However, this conceptualization of risk and intent does not reflect the multi-dimensional and dynamic nature of both risk and intent, which cover a wide continuous spectrum. Although the analyses followed clinical decision models that conceptualized current suicide risk and intent at the point of clinical assessment, it is good to bear in mind that accurate modelling of suicide risk requires using validated continuous measures of suicidal affect, behaviours and cognitions, incorporated into a comprehensive clinical assessment, as well as ongoing monitoring for fluctuations in risk and intent. Although the models demonstrated good clinical utility for our sample, the specific configurations of patterns of risk and protective factors might differ among individuals, further research to examine the intricacies of underlying patterns could be explored using in-depth interviews and qualitative methodology.

In conclusion, the findings have implications for informing best practice in suicide assessment and primary prevention for suicides in Singapore. By using brief risk assessments that are substantiated by empirical findings from current research in the local population, the clinician would be taking a step forward in utilizing the scientist-practitioner model in their evidence based practice. This study adds to the current literature on suicide risk and suicide intent, building evidence on the usage of recognized risk and protective factors in suicide assessment, and casting light on the relevance of prior planning and attempt to hide the suicide attempt in risk assessment protocols for use by emergency department clinicians following an index suicide attempt. The important contribution of habitual poor coping, serious financial problems, and expressed regret into suicide intent is highlighted and further enhances our understanding for how these factors could be incorporated in the formulation of intervention strategies in our efforts to prevent premature deaths by suicide in vulnerable individuals.

## References

[1] World Health Organization (2016). Global Health Observatory Data. Retrieved from: http://www.who.int/gho/mental_health/en/

[2] Langlois S., & Morrison P. (2002). Suicide deaths and suicide attempts. Health Rep 13:9–22. 12743953

[3] Beautrais A. (2007). Subsequent mortality in medically serious suicide attempts: A 5 year follow-up. Aust N Z J Psychiatry 37:395–599. 10.1046/j.1440-1614.2003.01236.x 14511088

[4] Linehan M., Comtois K.A., Brown M.Z., Heard H.L., & Wagner A (2006). Suicide Attempt Self-Injury Interview (SASII): Development, reliability, and validity of a scale to assess suicide attempts and intentional self-injury. Psychol Assessment 18:303–312. 10.1037/1040-3590.18.3.303 16953733

[5] Silverman M.M., Berman A.L., Sanddal N.D., O' Carroll P.W., & Joiner T.E. (2007). Rebuilding the tower of Babel: A revised nomenclature for the study of suicide and suicidal behaviours part 2: Suicide-related ideations, communications and behaviour. Suicide Life Threat Behav 37:264–277. 10.1521/suli.2007.37.3.264 17579539

[6] Cole D. (1988). Hopelessness, social desirability, depression and parasuicide in two college student samples. J Consult Clin Psychol 56:131–136. 3346438 10.1037//0022-006x.56.1.131

[7] Kral M., & Sakinovsky I. (1994). A clinical model for suicide risk assessment In Leenaars A., Maltsberger J., & Neimeyer R. (Eds.), Treatment of suicidal people (pp. 19–31). Washington: Taylor and Francis.

[8] Beck A., Weismann A., Lester D., & Trexler L. (1976). Classification of suicidal behaviors, II: Dimensions of suicidal intent. Arch Gen Psychiatry 33:835–837. 942287 10.1001/archpsyc.1976.01770070065006

[9] Beck A., Beck R., & Kovacs M. (1975). Classification of suicidal behaviours: Quantifying intent and medical lethality. Am J Psychiatry 132:285–287. 10.1176/ajp.132.3.285 1115273

[10] Linehan M. (1981). Suicidal Behaviors Questionnaire (SBQ). Seattle: University of Washington.

[11] Magaletta P.R., Patry M.W., Wheat B., & Bates J. (2008). Prison inmates characteristics and suicide attempt lethality: An exploratory study. Psychol Serv 5:351–361.

[12] Harris K. M., Lello O. D., & Willcox C. H. (2017). Reevaluating Suicidal Behaviors: Comparing Assessment Methods to Improve Risk Evaluations. *Journal of Psychopathology and Behavioral Assessment*, 39(1), 128–139. 10.1007/s10862-016-9566-6

[13] Beck A., Shuyler D., & Herman I. (1974). Development of suicide intent scales In Beck A., & Resnick D. (Eds.), The prediction of suicide (pp. 45–56). New York: Charles Press.

[14] Beck A., & Steer R. (1989). Clinical predictors of eventual suicide: A 5–10 year prospective study of suicide attempters. J Affect Disord 17:203–209. 2529288 10.1016/0165-0327(89)90001-3

[15] Silverman M.M., Berman A.L., Sanddal N.D., O' Carroll P.W., & Joiner T.E. (2007). Rebuilding the tower of Babel: A revised nomenclature for the study of suicide and suicidal behaviours part 1: Backgrounds, rationale, and methodology. Suicide Life Threat Behav 37:248–263. 10.1521/suli.2007.37.3.248 17579538

[16] Zalsman G., Braun M., Arendt M., Grunebaum M., Sher L., Burke A., Brent D., Chaudhury S., Mann J., & Oquendo M. (2006). A comparison of the medical lethality of suicide attempts on bipolar and major depressive disorders. Bipolar Disord 8:558–565. 10.1111/j.1399-5618.2006.00381.x 17042829

[17] Brown G., Henriques G., Sosdjan D., & Beck A. (2004). Suicide intent and accurate expectations of lethality: Predictors of medical lethality in suicide attempts. J Consult Clin Psychol 72:1170–1174. 10.1037/0022-006X.72.6.1170 15612863

[18] Lester D., & Beck A.T. (1975). Attempted suicide: Correlates of increasing medical lethality. Psychol Rep 37:1236–1238. 10.2466/pr0.1975.37.3f.1236 1208746

[19] Zhang J., & Xu H. (2007). Degree of suicide intent and the lethality of the means employed: A study of Chinese attempters. Arch Suicide Res 11:343–350. 10.1080/13811110701541889 17882622 PMC3210858

[20] King C. (1997). Suicidal behaviour in adolescence In Maris R., Silverman M., & Canetto S. (Eds.), Review of Suicidology (pp. 61–95). New York: Guilford.

[21] Neimeyer R., & Pfeiffer A. (1994). Errors in suicide intervention In Leenaars A., Maltsberger J., & Neimeyer R. (Eds.), Treatment of suicidal people (pp. 207–224). Washington: Taylor and Francis.

[22] Maris R., Berman A., & Maltsberger J. (1992). Summary and conclusions: What have we learned about suicide assessment and prediction In Maris R., Berman A., Maltsberger J. & Yufit R. (Eds.), Assessment and prediction of suicide (pp. 640–672). New York: Guilford Press.

[23] Beautrais A. (1998). Risk factors for serious suicide attempts among young people: A case control study In Kosky R., Eshkevari H., Goldney R., & Hassan R. (Eds.), Suicide prevention: The global context (pp. 267–282). New York: Plenum Press.

[24] Oquendo M., Currier A., Liu S., Hasin D., Grant B., & Blanco C. (2011). Increased risk for suicidal behaviour in comorbid bipolar disorder and alcohol use disorder. J Clin Psychia 71(7):902–909. 10.4088/JCP.09m05198gry PMC2914308 20667292

[25] Chia B. (1999). Too young to die: An Asian perspective on youth suicide. Selangor: Times.

[26] Nordstrone P., Asberg M., Asberg-Wistedt A., & Nordin C. (1995). Attempted suicide predicts suicide risk in mood disorders. Acta Psychiat Scand 92:345–350. 8619338 10.1111/j.1600-0447.1995.tb09595.x

[27] Vallente S., & Fann (2002). Overcoming barriers to suicide risk management. J Psychosoc Nurs Ment Health Serv 40:22–33. 10.3928/0279-3695-20020701-10 12136513

[28] Pokorny A. (1983). Prediction of suicide in psychiatric patients. Arch Gen Psychiatry 40;249–257. 6830404 10.1001/archpsyc.1983.01790030019002

[29] Goldney R., & Spence N. (1987). Is suicide predictable? Aust N Z J Psychiatry 21:3–4. 10.1080/00048678709160893 3476106

[30] Goldstein R., Black D., Nasrallah A., & Winokur G. (1991). The prediction of suicide. Sensitivity, specificity, and predictive value of a multi-variate model applied to suicide among 1,906 patients with affective disorders. Arch Gen Psychiatry 48:418–422. 2021294 10.1001/archpsyc.1991.01810290030004

[31] Furst J., & Huffine C. (1991). Assessing vulnerability to suicide. Suicide Life Threat Behav 21:329–344. 1799015

[32] Mann J. (2002). A current perspective of suicide and attempted suicide. Ann Intern Med 136:302–311. 10.7326/0003-4819-136-4-200202190-00010 11848728

[33] Chen V., Chou J., Hsieh T., Chang H., Lee C., Dewey M., … Tan H. (2013). Risk and predictors of suicide and non-suicide mortality following non-fatal self-harm in Northern Taiwan. Soc Psychiatry Psychiatr Epidemiol 48:1621–1627. 10.1007/s00127-013-0680-4 23563393

[34] Choo C., Diederich J., Song I., & Ho R. (2014a). Suicide Risk Analysis In Lech M., Song I., Yellowlees P., Diederich J. (eds). Mental Health Informatics. Springer: Berlin, Heidelberg New York (pp. 217–246).

[35] Choo C., Diederich J., Song I., & Ho R. (2014b). Cluster Analysis Reveals Risk Factors for Repeated Suicide Attempts in A Multi-ethnic Asian Population. Asian J Psychiatr 8:38–42. 24655624 10.1016/j.ajp.2013.10.001

[36] Chong M., Yeh E., & Wen J. (1992). Suicidal behaviour in Taiwan Kok, L., & Tseng, W. (1992). Orientation to cross-society comparison. In Kok L. & Tseng W. (Eds.), Suicidal behaviour in the Asia Pacific region (pp. 69–82). Kent Ridge: Singapore University Press.

[37] Judd F., Jackson H., Komiti A., Bell R., & Fraser C. (2012). The profile of suicide: Changing or changeable? Soc Psychiatry Psychiatr Epidemiol 47:1–9. 10.1007/s00127-010-0306-z 21052623

[38] Cheng A., & Lee C. (2000). Suicide in Asia and Far East In Hawton K. & Van Heeringen K. (Eds.), The international handbook of suicide and attempted suicide (pp. 29–48). West Sussex: John Wiley & Sons.

[39] LeardMann C., Powell T., Smith T., Bell M., Smith B., Boyko E., Hoge C. (2013). Risk factors associated with suicide in current and former US military personnel. JAMA 310:496–506. 10.1001/jama.2013.65164 23925620

[40] Chen Y. Y., Chien-Chang Wu K., Yousuf S., & Yip P. S. F. (2012). Suicide in Asia: Opportunities and challenges. Epidemiol Rev 34:129–144. 10.1093/epirev/mxr025 22158651

[41] Appleby L., & Turnbull G. (1995). Parasuicide in the first postnatal year. Psychol Med 25, 1087 8588005 10.1017/s0033291700037570

[42] Takahashi Y. (1998). Suicide in Japan: What are the problems? In Kosky R., Eshkevari H., Goldney R., & Hassan R. (Eds.), Suicide prevention: The global context (pp. 121–130). New York: Plenum Press.

[43] Evans E., Hawton K., & Rodham K. (2005). In what ways are adolescents who engage in self-harm or experience thoughts of self-harm different in terms of help-seeking, communication and coping strategies? J Adolesc 28:573–587. 10.1016/j.adolescence.2004.11.001 16022890

[44] Harris K. M., McLean J. P., & Sheffield J. (2013). Suicidal and online: How do online behaviors inform us of this high-risk population? Death Stud 38:387–394. 10.1080/07481187.2013.768313 24666145

[45] Schneidman E. (2001). Understanding suicide Washington: American Psychological Association.

[46] Kok L., & Tseng W. (1992). Orientation to cross-society comparison In Kok L. & Tseng W. (Eds.), Suicidal behaviour in the Asia Pacific region (pp.1–14). Kent Ridge: Singapore University Press.

[47] Bhugara D. (2002). Suicidal behaviour in South Asians in the UK. Crisis, 23:108–113. 10.1027//0227-5910.23.3.108 12542108

[48] Williams J.M., & Pollock L.R. (2000). The psychology of suicidal behaviour In Hawton K. & Van Heeringen K. (Eds.), The international handbook of suicide and attempted suicide (pp. 79–94). West Sussex: John Wiley & Sons.

[49] Kumar P. N. S., & George B. (2013). Life events, social support, coping strategies, and quality of life in attempted suicide: A case-control study. Indian J Psychiatry 55:46 10.4103/0019-5545.105504 23439644 PMC3574455

[50] Burón P., Jimenez-Trevino L., Saiz P. A., García-Portilla M. P., Corcoran P., Carli V., … Bobes J. (2016). Reasons for attempted suicide in Europe: Prevalence, associated factors, and risk of repetition. Arch Suicide Res 20:45–58. 10.1080/13811118.2015.1004481 26726966

[51] Stone D. M., Holland K. M., Schiff L. B., & McIntosh W. L. (2016). Mixed methods analysis of sex differences in life stressors of middle-aged suicides. Am J Prev Med 51:S209–S218. 10.1016/j.amepre.2016.07.021 27745609 PMC7068644

[52] de Bruine W., Dombrovski A. Y., Parker A. M., & Szanto K. (2016). Late-life depression, suicidal ideation, and attempted suicide: The role of individual differences in maximizing, regret, and negative decision outcomes: Maximizing, late-life depression, and suicide. J Behav Decis Mak 29:363–371. 10.1002/bdm.1882 27840559 PMC5100970

[53] Miller I. W., Camargo C. A., Arias S. A., Sullivan A. F., Allen M. H., Goldstein A. B., … Boudreaux E. D. (2017). Suicide prevention in an emergency department population: The ED-SAFE study. JAMA Psychiatry, 74(6), 563–570. 10.1001/jamapsychiatry.2017.0678 28456130 PMC5539839

[54] Stanley B., Chaudhury S. R., Chesin M., Pontoski K., Bush A. M., Knox K. L., & Brown G. K. (2016). An emergency department intervention and follow-up to reduce suicide risk in the VA: Acceptability and effectiveness. Psychiatric Services, 67(6), 680–683. 10.1176/appi.ps.201500082 26828397

